# Public Health Preparedness for and Response to Nuclear Disasters: An Editorial

**DOI:** 10.3390/ijerph15112489

**Published:** 2018-11-08

**Authors:** Shuhei Nomura, Michio Murakami

**Affiliations:** 1 Department of Global Health Policy, Graduate School of Medicine, The University of Tokyo, 7-3-1, Hongo, Bunkyo-ku, Tokyo 113-0033, Japan; 2 Department of Epidemiology and Biostatistics, School of Public Health, Imperial College London, Norfolk Place, London W2 1PG, UK; 3 Department of Health Risk Communication, Fukushima Medical University School of Medicine, 1 Hikarigaoka, Fukushima, Fukushima 960-1295, Japan; michio@fmu.ac.jp; 4 Radiation Medical Science Center for the Fukushima Health Management Survey, Fukushima Medical University, 1 Hikarigaoka, Fukushima, Fukushima 960-1295, Japan

In 2011, resilience to nuclear disasters emerged as a core public health challenge. Japan’s Fukushima nuclear disaster in 2011 not only showcased fundamental weaknesses in the country’s preparedness and responses to nuclear emergencies, but also highlighted the importance of focusing more attention on the management of nuclear disasters—at individual, community, and policy levels—in global disaster debates [1]. The challenges of the Fukushima disaster have shown that national and global policies on nuclear disaster management are in urgent need of reform and reinforcement.

A nuclear disaster is very complex and not necessarily comparable to other types of natural or man-made disasters. This is because of its serious social consequences for the human security (e.g., health and wellbeing) of present and future generations, as well as the environmental consequences due to the associated release of invisible, odourless, and long-lived radioactive materials. Due to possibly excessive radiation levels at a major disaster site, preferred emergency measures may not always be available or feasible and may also be limited due to the regional topography and the meteorological situation. For these reasons, the prime aim of this special issue is to inform the design, preparation, and delivery of measures (including public risk communications) to advance effective countermeasures for the recovery of affected areas in the aftermath of past or ongoing nuclear disasters including the Fukushima disaster, and to manage future major nuclear disasters by adding empirical evidence. Thus, we can move towards reaffirming that “never again will we have another Fukushima disaster”. This collection of papers should be immensely useful for readers including researchers, policymakers, practitioners, and professionals.

In July 2017, this special issue was initiated by calling for papers from diverse disciplinary backgrounds. A significant number of scholars responded and a total of 22 manuscripts were submitted. Through a single-blind review process following standard MDPI review guidelines, we invited at least 37 expert reviewers (after a preliminary editorial judgement for peer-review) to review the manuscripts and comment on the quality, originality, relevance, as well as fit for the special issue. This led to 14 of the 22 submitted manuscripts being accepted (63.6% acceptance rate) for publication in the special issue.

The special issue consists of 2 review papers and 12 original articles, all of which address the Fukushima disaster. Out of them, nine studies (64.3%) investigated radiation risk perception (e.g., anxiety about adverse health effects of radiation exposure post-incident) or psychological morbidity in the post-emergency phase of the disaster.

Takebayashi et al. performed a systematic review of the literature published between March 2011 and 16 May 2017 on radiation perception and anxiety after the Fukushima disaster [2]. They summarized that the governing factors of radiation risk perception included demographics, disaster-related stressors, trusted information, and radiation-related variables; and that the effects of radiation risk perception comprised severe distress, intention to leave employment or not to return home, or other dimensions. Miura et al., Orui et al., Oe et al., Itagaki et al., Suzuki et al., Kuroda et al., and/or Ito et al. added new findings to the systematic reviews of Takebayashi et al. [3,4,5,6,7,8,9]. They demonstrated that the Fukushima disaster imposed various dimensions of insufficient physical activity, inappropriate sleep, and psychological health risks among affected people. In particular, radiation perception and anxiety were strongly associated with other mental disorders, highlighting the unique severity of a nuclear disaster. Orui et al. also suggested that those who laughed frequently, had a social network, and felt satisfied with their working and living environments, were more likely to maintain psychiatric stability [4]. Murakami et al. assessed the effects of various radiological countermeasures on subjective well-being and mental health conditions post-disaster. Thyroid examination was associated with not only a reduction in anxiety but also an increase in stress. Those who participated in food inspection showed a lower improvement in self-rated health. Those who attended some kind of explanatory meetings (where authorities or professionals explained the situation with regard to radiation exposure to the local residents) showed increased sadness, worry, and anxiety [10].

Two studies, Uchiyama et al. and Murakami et al., added evidence on internal radiation dose assessment for the inhalation of radio-iodine and/or ingestion of radio-cesium. The authors emphasized that by taking into account adequate exposure scenarios, internal dose assessment based on environmental monitoring or survey data could yield good reliability, equivalent to the methods using direct measurement, such as anthropogammametry [11,12]. Murakami et al. also proposed a greater use of their assessment approaches and methodologies in a range of food regulatory measures for a future nuclear disaster.

Kuroda et al. highlighted the reduced functional activity among elderly evacuees after the Fukushima disaster, and proposed this be considered when developing resilient capacities in disaster preparedness [13]. Hasegawa et al. stressed that by promoting social capital (e.g., social networks, reciprocal ties, social participation, etc.), people become more likely to engage in disaster-preparedness activities [14]. Through rigorous literature reviews including media reviews, Sato and Lyamzina warned that a heavy focus solely on long-term environmental contamination might divert attention from local people’s perceptions about radiation risks, which might delay or even make the recovery process more complex [15].

In conclusion, these papers demonstrate an elevated understanding of the perception of radiation risks and psychological distress following major nuclear disasters. They also indicate the potential latitude for improving the evaluation of internal radiation exposure. Furthermore, the importance of social capital in emergency preparedness was demonstrated. Overall, this special issue has successfully added numerous findings for reducing post-disaster morbidity by better preparedness. Unexpectedly, there were no accepted manuscripts dealing with pre-Fukushima nuclear disasters (e.g., the 1945 atomic bombing of Hiroshima and Nagasaki, the 1979 Three Mile Island accident, the 1986 Chernobyl disaster, etc.) in exploring the landscape of public health preparedness and responses to nuclear disasters. This selection, however, addresses a broad range of aspects, leveraging the multidisciplinary vision of nuclear disaster research.

We stand at the crossroads of deciding how to go about building our future nuclear disaster resilient societies. Nuclear risk is not only an issue facing Japan. The 25 oldest nuclear reactors in Europe are now close to, or past their, originally envisioned 40 years of operation [16], and their vulnerability to failure and accidents has grown, with a reported 50% increase in unexpected incidents between 2000 and 2006 [17]. Also, more than two-thirds (74 of 100) of the operating nuclear reactors in the United States have received extended licences permitting 60 years of operation, far beyond their original design lifetimes of 40 years [16,18]. As a result, we are entering a new era of nuclear risk. In this context, it is vital that we learn from the challenges posed by Japan’s Fukushima nuclear disaster to build resilient capacities at all levels (individual, community, and policy) for preparedness and response to future nuclear crises, globally. We hope that the implications for practice derived from the special issue will be carefully considered at all levels and promote discussion about the successful implementation of effective preparedness and responses to the risk of nuclear crises.
